# Multi-Objective Distributed Client Selection in Federated Learning-Assisted Internet of Vehicles

**DOI:** 10.3390/s24134180

**Published:** 2024-06-27

**Authors:** Narisu Cha, Long Chang

**Affiliations:** 1 The School of Computer and Information Management, Inner Mongolia University of Finance and Economics, Huhort 010051, China; 2 The School of Statistics and Mathematics, Inner Mongolia University of Finance and Economics, Huhort 010051, China; changlong@imufe.edu.cn

**Keywords:** federated learning, internet of vehicles, distributed client selection, multi-objective fuzzy evaluator

## Abstract

Federated learning is an emerging distributed machine learning framework in the Internet of Vehicles (IoV). In IoV, millions of vehicles are willing to train the model to share their knowledge. Maintaining an active state means the participants must update their state to the FL server in a fixed interval and participate in the next round. However, the cost of maintaining an active state is very large when there are a huge number of participating vehicles. In this paper, we propose a distributed client selection scheme to reduce the cost of maintaining the active state for all participants. The clients with the highest evaluation are elected among the neighbors. In the evaluator, four variables are considered, including the sample quantity, available throughput, computational capability, and the quality of the local dataset. We adopt fuzzy logic as the evaluator since the closed-form solution over four variables does not exist. Extensive simulation results show that our proposal approximates the centralized client selection in terms of accuracy and can significantly reduce the communication overhead.

## 1. Introduction

Over the past years, federated learning (FL) [1,2] has become an emerging distributed machine learning framework that has received widespread attention from academia and industry. It can achieve the purpose of exchanging knowledge across users by sharing training models while avoiding the leakage of privacy, as it does not require uploading raw data related to user privacy to the server. Enabling federated learning in the Internet of Vehicles (IoV) [3,4] serves as an application case, not only protecting the privacy and user concerns but also increasing computational efficiency by distributed parallellism over the clients, utilizing the idle resources in vehicular networks. In this framework, millions of vehicles with local datasets are eager to participate in training. For example, in GBoard (a keyboard developed by Google Inc. for tablets and smartphones), over 1.5 million clients are involved in the language model learning process through federated training [5]. Similarly, in FL-assisted IoV, there are over 3 million active vehicles in the Kanto area, Japan. As considered in [3], in venues like concerts or sports events, precious wireless resources would become more scarce when all nodes (vehicles or phones) in such crowded scenarios participate in federated learning, further affecting the learning efficiency. Due to wireless bandwidth limitations, only a small fraction of users are selected as clients for learning in each round. In the classical federated learning framework, the server is responsible for client selection. To better improve overall performance, which variables to consider in the selection process is still under debate. Therefore, all participants willing to train the model must periodically update all active states. As mentioned in [5], user devices must meet specific conditions, including dataset freshness, size, charging status, idle wireless connection, operating system version, and hardware requirements. Only considering the number of CPU cores, memory, and storage capability in heterogeneous FL, while neglecting the accuracy and loss of the model, is insufficient when selecting nodes [4]. This approach has led to algorithms being unable to handle non-independent and identically distributed datasets effectively. In FL-assisted IoV, vehicle states such as location, network environment, velocity, acceleration, and dataset quality vary over time, requiring prompt updates to the server over a wireless connection. Compared to model uploading, the overhead of continuously updating the state from all participants to the server is very large, given the large number of participants.

Most classical federated learning frameworks adopt a centralized control scheme, where the server selects clients for learning. The server collects and updates the active state of all participants, choosing a set of participants with active states as training clients at the beginning of each communication round. Undoubtedly, the overhead from state updates is substantial when dealing with a large number of participants. These overheads consume precious wireless resources, increase communication delays, and often result in inefficiencies. For example, the resources in the wireless network cannot sustain the connection of so many nodes participating in FL [3]. As concluded in [6], achieving good performance can also be accomplished by selecting only a small fraction of devices in each round. Unfortunately, many researchers have not addressed the overhead associated with updating the state of all participants; instead, they have focused on frameworks that adapt decentralized client selection by updating the active state of all participants.

To address the issue of excessive information exchange, the server transfers the responsibility of client selection to the clients, no longer receiving participant information for evaluation. Meanwhile, the assessment of the node shifts from the server to the local. Each participant assesses themselves based on input parameters such as sample quantity, network throughput, computational capability, and diversity of the local dataset.

According to the description of the centralized selection process in [6,7], the fundamental differences between centralized client selection and distributed client schemes are listed below: firstly, whether information from all participants is collected for evaluation on the server side, and secondly, whether the server has the authority to select clients. Furthermore, participant assessment is conducted locally rather than on the server. Most traditional federated learning frameworks are developed under centralized schemes, resulting in significant overhead from information updates that cannot be eliminated. Conversely, distributed frameworks with client selection effectively eliminate this overhead.

Notice that the model and exchanged data are not the same. Exchanged data include the state information of the vehicle, such as position, available bandwidth, data samples, etc., which vary over time. These data play a key role in selecting the clients and need to be updated in real time [6,7]. Additionally, these data compete for wireless resources with the model when a centralized scheme (where all information is transmitted to the base stations (BSes) or the central server) is adopted. To reduce resource competition, a distributed scheme using dedicated short-range communication (DSRC) technology, such as data exchanged using IEEE 802.11p, is a better option [3]. Our goal is to reduce competition between the model and vehicle data through an isolated transmission channel. Under this consideration, a distributed scheme may be better than a centralized scheme. Meanwhile, the distributed scheme results in less communication overhead compared to other schemes as well.

In federated learning-assisted IoV, selecting vehicles with high performance in each round can significantly accelerate model convergence. For instance, a client with a larger training dataset, stronger computational capability, higher network throughput, and local dataset with higher diversity (equivalent to higher loss function) ensures successful uploading of the client model with more knowledge to the central server. Consequently, global model accuracy improves as more local models participate in aggregation [8]. However, there is no closed-form solution that comprehensively considers all influencing factors, posing a significant challenge in evaluating participating vehicles. To address this challenge, we propose a lightweight evaluation approach: fuzzy logic-based evaluator, which leverages fuzzy relationships between influencing factors to construct a participant assessment approach.

In conclusion, two research gaps in the existing studies have not yet been fully investigated: Firstly, the exchange of state information between the central server and all participants becomes significant as the number of participants in the centralized framework grows very large. Secondly, there is no closed-form solution for assessing nodes, especially when considering multiple variables. To this end, we propose a distributed client selection framework with fuzzy logic. The main contributions of the paper are outlined as follows:

- A distributed client selection framework is proposed. In this framework, to reduce the exchange of state information between candidate nodes and the server, the server no longer has the authority to select clients, and state information is not updated to the central server. The selection process is performed at each node.
- A multi-objective client evaluator based on fuzzy logic is proposed to address heterogeneity in federated learning. This evaluator considers four objectives: sample quantity, available throughput, computational capability, and the loss function of the local dataset. Moreover, the evaluator operates locally instead of on the central server, as the central server does not collect client information.
- A simulator combined with realistic vehicular networks and federated learning is constructed to validate the proposal. Additionally, we synthesize a non-independent and identically distributed dataset with varying levels of heterogeneity to demonstrate the superiority of our framework under heterogeneous dataset conditions.

The remainder of the paper is organized as follows: Section 2 summarizes significant existing works. Section 3 introduces the system model including network architecture, federated learning framework, and FL-assisted IoV. Section 4 and Section 5 elaborate on the distributed client selection framework and the multi-objective client evaluator, respectively. Section 6 discusses the simulator setup and experimental results. Finally, Section 7 concludes the paper.

## 2. Related Works

Over the past years, the research gap in the exchange of state information between the FL server and the vehicle has been ignored. Most researchers have focused on addressing the bottleneck in communication costs between the server and clients for updating models in centralized FL [9,10,11,12,13], while our advanced proposal primarily focuses on a distributed framework to eliminate this information exchange. Unlike the centralized schemes proposed below, such as gradient and model compression [9,10], biased client selection [6,11,14], reinforcement learning-based client selection [15,16], learning on the edge [12], and hierarchical federated learning [17], we explore a distributed framework where the server does not select the clients. The federated learning frameworks mentioned above employ centralized client selection, where state information about participants must be gathered by the FL server to maintain their active state, and the FL server handles the client selection process. In fact, millions of nodes are willing to participate in federated learning training. For example, millions of vehicles can join training in IoV. Typically, only a small number of vehicles are selected as clients [6]. In such scenarios, the communication overhead from maintaining the active state of all participants exceeds the overhead from updating the model between the FL server and clients. Reducing the exchange of state information is crucial in FL for saving bandwidth. Unfortunately, this issue has not been fully investigated.

The decentralized federated learning (DFL) framework has been widely discussed; however, it differs from our proposal. In DFL, there is no aggregating server required, and each client’s training model is sent to all other clients. Due to the lack of client selection, not only does the communication cost increase significantly but the robustness is also a concern in large-scale scenarios. The DFL based on peer-to-peer communication for medical applications proposed in [18] performs well with fewer nodes. Another approach, presented in [19], utilizes a DFL framework with a committee consensus blockchain, storing all models, including the global model from the server and local models from clients. Similarly, ref. [20] introduced an asynchronous DFL framework based on blockchain to enhance stability and reliability in model transmission within IoV. Additionally, the authors in [21] proposed decentralized federated learning based on blockchain specifically for vehicular networks, and analyzed its advantages theoretically. Meanwhile, unreliable networks and updating model are also taken into consideration in [22]. The model will be fully updated when the connection state is good. Otherwise, the model will only be partially updated when the connection state is noisy. The DFL frameworks mentioned above are particularly suitable for dynamic network environments such as vehicular networks with mobility. Unfortunately, these distributed frameworks do not consider the overhead of exchanging state information. The distributed scheme in our proposal divides all vehicles into multiple small cells based on geographical position, and the information exchange among vehicles uses DSRC technology.

In contrast to the centralized client selection framework, distributed client selection does not involve the server gathering all participant information. Client evaluation and selection are removed from the server’s responsibilities. Client selection is classified as biased or unbiased. Unbiased client selection, such as random selection [6], gives all participants an equal chance of being selected. Biased client selection selects clients based on specific criteria. In [23], client selection is systematically summarized, involving opportunities and challenges, and emphasizing the importance of the heterogeneity. In [24], the authors investigated existing works related to system architecture, applications, privacy concerns, and resource management in federated learning. In [25], the authors provided a taxonomy and highlighted the challenges of client selection in terms of fairness to promote sustainability in the FL ecosystem. Likewise, ref. [26] developed a novel global model aggregation algorithm focusing on group fairness rather than the weight related to sample quantity in local models. Furthermore, ref. [14] selected clients based on larger loss functions to accelerate convergence. Ref. [27] jointly considered wireless resource allocation and long-term client selection perspectives. Ref. [7] incorporated multiple criteria like computational capability, memory, and energy in client selection to maximize model upload success rates. In contrast, ref. [20] proposed asynchronous federated learning, where deep reinforcement learning (DRL) on the server selects nodes with higher communication and computation resources for training, uploading local models to the blockchain instead of the server. Finally, in aggregation, the server retrieves global models from the blockchain after local training. Ref. [28] considered client selection from efficiency and fairness perspectives. Despite these advancements, challenges persist in complex networks like federated learning-assisted IoV due to client heterogeneity. In [8], the authors proposed client selection considering multi-objective evaluation in the centralized client selection framework, failing to decrease the overhead of exchanging state information. Heterogeneity is one of the key challenges in the FL, encompassing statistical and system variations that reduce accuracy and slow convergence. To address statistical heterogeneity, FedProx [29] introduced a proximal term in local objective functions to minimize gradient drift, yet did not address system heterogeneity. For example, vehicle mobility in IoV can disrupt connections. Ref. [30] jointly considered node selection and wireless resource allocation in heterogeneous FL systems to maximize loss function decay and accelerate convergence. Addressing non-independent and identically distributedness, the authors in [31] employed support vector machines (SVMs) to detect sample features and eliminate irrelevant samples. SCAFFOLD [32] adjusted update directions by comparing global and local models. While these studies provide theoretical and methodological insights, their integration into real-world applications remains limited. Unfortunately, the mentioned works above have not proposed a selection approach involving multiple factors, such as heterogeneity and bandwidth etc.

Previous works did not consider a research gap, the exchange of state information between clients and servers, which is essential for node selection. The state information exchange becomes huge when the number of candidate nodes participating in training is large. Additionally, the heterogeneity is a key challenge in federated learning. Considering heterogeneity among nodes during the selection process can greatly enhance the model accuracy and accelerate the convergence. To address the issues above, we propose a distributed client selection scheme, which leverages two different channels, specifically, the cellular networks to transmit the model between vehicles and the central server, and DSRC technology to exchange the state information between vehicles in the vicinity. The proposal selects the clients by distributed approach, like each individual votes independently by in a election. To sort the all participants, a scalar value is required for the assessment of vehicle. Multi-objective evaluator is adopted to assess heterogeneous nodes with varying computational capability, communication bandwidth, sample quantity, and data with non-independent and identically distributedness.

## 3. System Model

In this section, we firstly introduce the system model used in the paper. Then, the framework of federated learning is described briefly. Finally, federated learning-assisted IoV is presented.

### 3.1. Network Architecture

**Figure 1:**
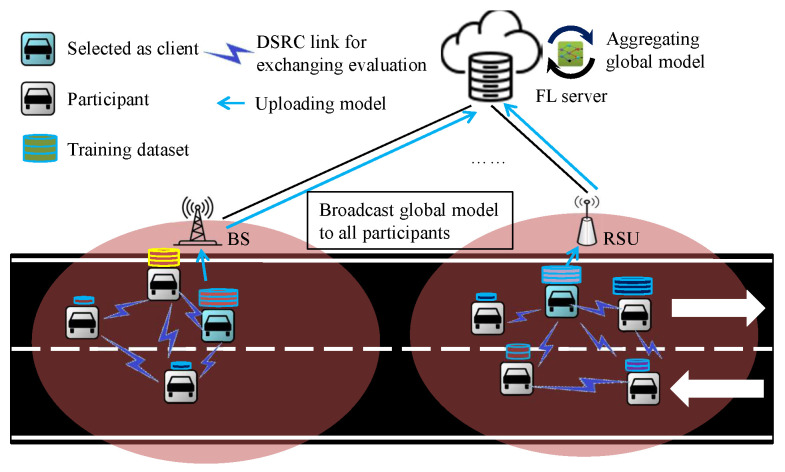
Realistic scenario using distributed client selection framework.

We consider an urban scenario where cellular networks are employed for communication. Each vehicle also supports DSRC technology to exchange evaluations with neighbors as shown in Figure 1. Evaluations are assessed locally by a multi-objective evaluator for each vehicle. Millions of participating vehicles are running in the long straight road and covered by multiple BSes. These BSes share the FL server deployed in the cloud. FL server only maintains a global model in each round. All vehicles move uninterruptedly from one BS to another. The throughput availability of the vehicles varies with the movement. We assume that each participating vehicle is willing to join the learning process and share its knowledge with FL and other vehicles.

Let Φ denote the set of participating vehicles indexed by *i*. Vehicle $P_{i}\in \Phi$ possesses available computational resources as well as training samples $D_{i}^{k}=\left(x_{i}^{k},y_{i}^{k}\right)$, where $x_{i}^{k}$ refers to data of a sample and represents as a vector, $y_{i}^{k}$ refers to the label of a sample. Let $C_{i}$ denote the computational capability ratio of vehicle $P_{i}$. Considering that the quantity of samples varies with time, the quantity of samples in vehicle $P_{i}$ in the round *k* is denoted as $|D_{i}^{k}|$. $|D^{k}|$ denotes the sum of the samples across all clients in the round *k*. Each BS can schedule wireless resource blocks (RBs) to allocate clients for model transmission. An X2 interface connects neighboring BSes to support efficient handover. The wireless resource block allocation strategy assumes independence and does not interfere with others. BSes allocate RBs using “MAX C/I” (Maximum Carrier-to-Interference) scheduling when multiple clients apply simultaneously.

### 3.2. Federated Learning

Federated learning is a distributed machine learning paradigm in which user data are kept local during the learning to protect the user’s privacy concern. In the classical federated learning [1], the whole process in each round is divided into four steps: broadcast global model, local updates over local data, upload local model and aggregation global model for the next round.

Each client updates their local model over local data according to the equation below:

(1) $$w_{i}^{k}=w_{i}^{k}-\eta \frac{\partial L_{i}\left(w_{i}^{k}\right)}{\partial w_{i}^{k}},$$

where η is referred to as the learning rate. $w_{i}^{k}$ and $L_{i}\left(w_{i}^{k}\right)$ are referred to as the local model and the loss function of the client *i* in round *k*, respectively. In general, the boldface of variables denotes the tensor, like model parameters. The local model is uploaded to the server for aggregation after the training.

The global model for the next round is generated on the server by the equation below:

(2) $$w_{g}^{k+1}=\sum_{i=1}^{N}\frac{\left|D_{i}^{k}\right|}{\left|D^{k}\right|}w_{i}^{k}.$$

Global loss function is defined by the equation below:

(3) $$L_{g}\left(w_{g}^{k+1}\right)=\sum_{i=1}^{N}\frac{\left|D_{i}^{k}\right|}{\left|D^{k}\right|}L_{i}\left(w_{i}^{k}\right),$$

where *N* refers to the number of selected clients in round *k*. $\left|D^{k}\right|=\sum_{i}\left|D_{i}^{k}\right|$. $w_{g}^{k+1}$ is referred to the global model in round *k* + 1. In general, the symbol |x| denotes the number of the elements in set *x*. The categorical cross-entropy loss is adopted to output the probability of the multi-classification in the local training:

(4) $$\min_{w}L_{g}\left(w\right)=\min_{w}\sum_{i=1}^{N}\frac{\left|D_{i}^{k}\right|}{\left|D^{k}\right|}L_{i}\left(w_{i}^{k}\right).$$

### 3.3. Federated Learning Assisted IoV

The critical challenges of intelligent transportation systems (ITSs) are system heterogeneity, model performance, and user privacy [33]. System heterogeneity refers to the resources, such as computational capability, available network resources, and training datasets owned by each vehicle running on the road, which differ from other vehicles. In general, these resources vary with the vehicle state as well. For example, a vehicle parking on the lot has more resources compared to a vehicle driving fast on the highway in terms of the computational capability and training dataset. In the same way, the available network resources vary with vehicle movement, while the vehicle moves in and out continuously from the signal coverage of the roadside unit (RSU) located on the side of the road. The model performance worsens drastically when the network environment changes with the vehicle’s mobility. The model having stable and high safety is important for safeguarding passengers and pedestrians. The privacy concern for the vehicle, such as trajectory and traffic context, not only affects the driving experience but it can even endanger the life of pedestrians.

Federated learning is realized as an emerging effective maneuver for protecting user privacy and can be applied to the IoV regarding data sharing, collaborative intelligence, and distributed machine learning [20,34]. In the future, vehicles with intelligence will be mainstream in the ITS, in which various AI apps are deployed to assist with driving. Additionally, the vehicle has some resources such as computational capability, communication ability, and storage for processing the data collected by the sensors. These vehicles not only exchange basic information with each other but also share the knowledge learned from the AI model among the vehicles.

## 4. Distributed Client Selection Framework

In this section, we firstly present the workflow of client selection in the distributed scheme. Then, the differences between centralized federated learning (CFL) and distributed federated learning are discussed. Three different client selection schemes are compared, and the advantages of the distributed client selection scheme are described. Following that, we present two types of communication overhead in federated learning: the overhead from exchanging models between the FL server and the clients, and the overhead from maintaining the active state of all participants. We also compare these two types of overhead using GBoard as an example. Finally, we describe the distributed client selection in detail.

### 4.1. Workflow of Distributed Client Selection

**Figure 2:**
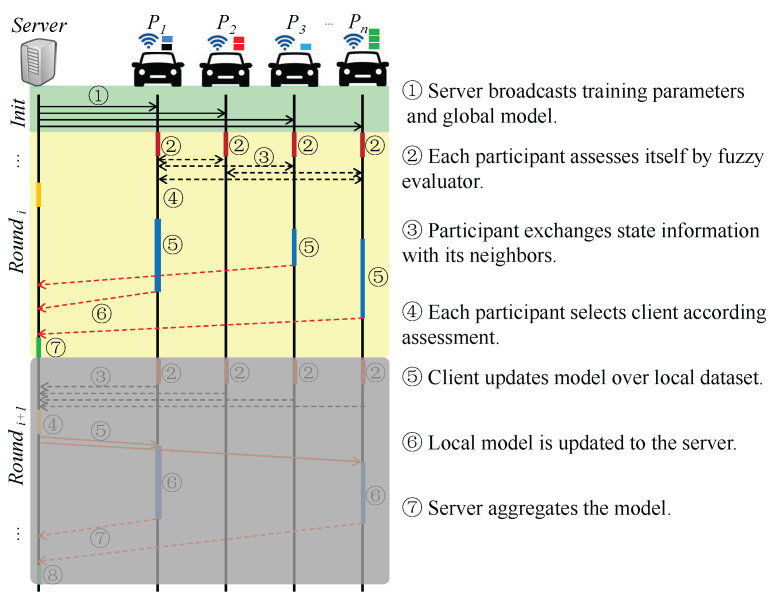
The workflow of distributed client selection with fuzzy evaluator.

The workflow of the distributed client selection scheme is presented below as shown in Figure 2. The selection process does not require the state information gathered in the server, including the assessment of nodes calculated by the fuzzy evaluator. Each communication round consists of seven steps, which can be grouped into two stages: the selection stage and the training stage. Steps ➀, ➁, ➂, and ➃ belong to the selection stage, while the remaining steps belong to the training stage.

Selection stage: The server is responsible for broadcasting fuzzy parameters involving the membership functions and the fuzzy rules to all participants at the beginning of the communication round. The vehicles assess themselves according to the current state information. Then, the assessment is exchanged with the neighbors. Finally, each participant sorts and selects the clients from the top *m* neighbors as the clients. The fuzzy parameters are configured based on historical records stored on the server. It is assumed that the server can analyze the historical records and find the optimal fuzzy parameters to meet the system’s requirements. This stage corresponds to steps ➁–➃ in Figure 2. Notably, step ➀ is run only once at the initialization of the FL.

Training stage: In this stage, every client updates their model over the local dataset. The local model is uploaded to the server once the training is finished. The server aggregates the local models to generate a new global model for the next round when the wall clock elapses. This stage corresponds to steps ➄–➆ in Figure 2.

### 4.2. Distributed Client Selection Framework

In CFL, the FL server collects local models from the clients and then aggregates them into a global model using the federated averaging algorithm (FedAvg) [1]. Meanwhile, all participant states are collected by the FL server for client selection. Conversely, in DFL, the server’s functions are distributed across all clients, including broadcasting the model, uploading the model to other clients, and aggregating the model. Thus, the server’s role is eliminated in FL. Compared with CFL, DFL is more suitable for dynamic networks and offers better scalability and robustness. However, in DFL, there is a potential waste of network resources because models are transmitted multiple times among the clients.

**Figure 3:**
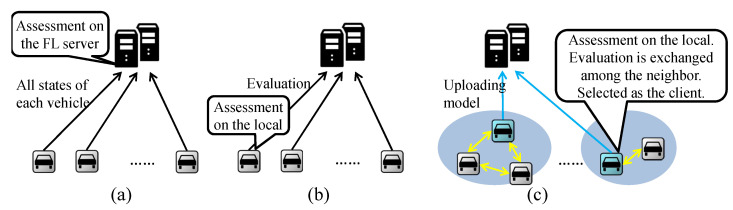
Different client selection schemes in FL. (**a**) Client selection in the CFL. (**b**) Client selection in the CFL-fuzzy [8]. (**c**) Distributed client selection.

Next, we discuss three client selection schemes as shown in Figure 3: client selection in CFL, client selection in CFL-fuzzy [8], and distributed client selection. In CFL, the states of each vehicle are collected by the FL server, which then performs assessment, sorting, selection, broadcasting the global model to all clients, local training, uploading local models, and aggregation as shown in Figure 3a. The FL server in CFL acts as the coordinator. For client selection in CFL-fuzzy, the assessment of each participant is processed locally and then updated to the FL server. Subsequently, the steps of sorting, selection, broadcasting the global model to all clients, local training, uploading local models, and aggregation are carried out as shown in Figure 3b. In distributed client selection, the global model is broadcast to all participants at the start of each round, and the assessment is processed locally. Then, evaluations are exchanged among neighbors, and clients are selected. The selected clients train the model on their local dataset and upload their local models to the FL server. Finally, the FL server aggregates all local models received from the clients as shown in Figure 3c. The detailed process is presented in Section 4.4. The characteristic of distributed client selection is that client selection adopts a distributed scheme, while model aggregation adopts a centralized scheme. Furthermore, the FL server does not know which participants are selected as clients. This scheme minimizes the overhead caused by maintaining the active state of all participants while maintaining the high efficiency of centralized aggregation.

Adopting the advantages of the distributed client selection scheme, the wireless resource competition between the model and state information can be avoided by using two different channels, specifically, the cellular network and DSRC technology, to transmit the model and the state information, respectively. Conversely, DSRC technology fits well with the distributed scheme, as the vehicles in the vicinity can form a group and select the client. Additionally, the multi-objective evaluator can work well independently, only receiving information from the central server, such as the maximum value of historical bandwidth. Then, the evaluation of vehicles is exchanged among the neighbors without being updated to the central server. For more details about the multi-objective evaluator, please see Section 5.

Considering the reproducibility of the proposal, firstly, some variables remain constant, such as the maximum network bandwidth, the maximum computational capability of the vehicle, and the maximum training data volume on the vehicle. For example, the maximum network bandwidth is typically related to the capacity of infrastructure like RSU. RSU are upgraded every decade or even longer, which can be considered constant for federated learning. Therefore, federated learning in vehicular networks only needs to consider the specific values of each variable in time to evaluate the assessment of the vehicle, continuously providing data for node selection in the distributed scheme. Secondly, DSRC technology, as a short-range communication technology, has a communication range among vehicles mainly determined by the transmitting power and frequency. Hence, the proposal can operate robustly in any scenario without requiring additional settings. Thirdly, the multi-objective evaluator employs fuzzy logic, which can easily reconfigure the weight between different variables. Meanwhile, removing or adding variables is also easy, making it adaptable to any scenario.

### 4.3. Communication Overhead

In the FL system, the communication overhead comprises two parts, the overhead by exchanging models and the overhead by maintaining the active state of all participants. In the dynamic network (e.g., IoV), the participant’s state needs to be constantly updated to the coordinator, such as the FL server, because the resources vary with time continuously. Notice that the participants send a message presenting “I have alive” to the coordinator. The state changes may increase the chance of being selected as a client. So, all participants update the active state to be chosen as a client by the server. In general, the size of maintaining an active state is far larger than the size of exchanging the model when millions of participants exist in the FL system. Next, we analyze a real example from Gboard [5] to compare the two kinds of overhead. We choose the transmitted data size as a comparing metric. Here, *N* is the number of all participants, and τ denotes the interval of sending state. *s* denotes the size of the state, which includes participant id, resource information (e.g., computational capability, available network throughput, and sample quantity), and other information (e.g., vehicle position, acceleration, and energy). *t* denotes the length of a communication round. *m* denotes the size of the model. The transmitted data size for maintaining the active state of all participants is defined by (5):

(5) $$c=\frac{stN}{\tau }$$

The parameters referred from [5] are listed in Table 1.

**Figure 4:**
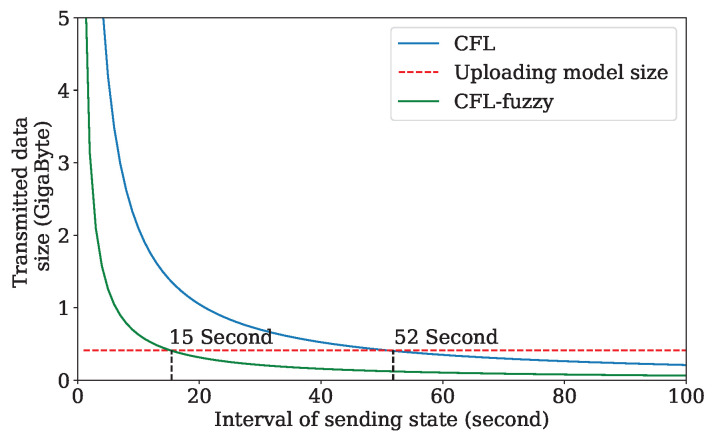
Comparison of two kinds of overhead. The dashed red line presents the size of the uploading model in each round. The blue and green lines present the overhead by maintaining the active state for all participants in CFL and CFL-fuzzy, respectively.

We compare with CFL and CFL-fuzzy [8] in terms of the overhead maintaining an active state for all participating devices in the Gboard. The dashed red line represents the size of the uploading model in each round over on the selected 300 client devices. The observation from Figure 4 is that the size of overhead maintaining the active state of all participating devices reaches 22.5 gigabytes in the interval of 1 s. In comparison, the uploading model size is only 0.41 gigabytes. The size of maintaining the active state of all participants decreases with the interval increase. Two curves, CFL and CFL-fuzzy, are crossed with the uploading model size curve at 52 s and 15 s, respectively. However, in a dynamic network, such as IoV, some clients with poor performance are selected and dropped down the model convergence and even cause traffic accidents because the state of the vehicles cannot be updated in such an interval. The distributed client selection framework proposed can achieve low communication overhead and the updating interval of the active state.

### 4.4. Distributed Client Selection Algorithm

We considered how much a client contributes to the global model in this framework. The client with a larger loss function has more contribution. So, the loss function of the dataset is introduced and used as one of the input variables in the multi-objective evaluation. To this end, the global model is broadcast to all participating vehicles at the start of each round. Every vehicle calculates the loss function without updating the model locally. Algorithm 1 presents the whole process in each round.
**Algorithm 1** Distributed client selection algorithm.**FL server:**1:$w_{i}^{k+1}\leftarrow w_{g}^{k},i=1,2,\ldots ,N$                          ▹ Broadcast global model to all clients.2:**while** The deadline is not expired **do**3:    Store $w_{i}^{k+1}$ from client *i*.4:**end while**5:$w_{g}^{k+1}\leftarrow \sum_{i=1}^{N}\frac{\left|D_{i}^{k+1}\right|}{\left|D^{k+1}\right|}w_{i}^{k+1}$**Each participant $P_{i}$:**1:$L_{i}\leftarrow \frac{\sum_{i=1}^{|D_{i}^{k}|}L_{i}\left(w_{i}^{k},x_{i}^{k},y_{i}^{k}\right)}{|D_{i}^{k}|}$                                 ▹ Calculate loss and no updating model.2:Evaluate the participant $E_{i}$.3:**if** $E_{i}$≥$E_{\tau }$ **then**                                              ▹$E_{\tau }$ is constant and used as a threshold.4:    $E_{i}$ is broadcast to the neighbors.5:**end if**6:**if** $E_{i}$ is the largest among the neighbors **then**7:    $P_{i}$ is a client.8:    $w_{i}^{k+1}\leftarrow w_{i}^{k+1}-\eta \frac{\partial L_{i}\left(w_{i}^{k+1}\right)}{\partial w_{i}^{k+1}}$9:    Uploading model to the FL server.10:**end if**

Notably, the broadcasting of the model in the distributed client selection involves transmitting it to all participating vehicles. Technically, reliable broadcasting has not yet been achieved. Fortunately, transmitting the model does not necessarily require reliable transmission, as federated learning (FL) can tolerate errors in the model parameters during the transmission stage. Therefore, broadcasting can be implemented using technologies such as multi-cast streaming.

## 5. Multi-Objective Evaluator

In this section, we describe indispensable parts of the evaluator, including the prediction of the available network throughput and the time taken for training. We present four input variables for the fuzzy evaluator. Next, we explain the fuzzy evaluator, which includes fuzzy rules, the normalization of input variables, and final evaluation. Finally, we illustrate the process of exchanging evaluations.

The evaluator is another important component of distributed client selection, running on each participating vehicle. In the evaluator, we consider four variables—sample quantity, network throughput, computational capability, and loss function of the local dataset—all obtainable locally. Due to the absence of a closed-form solution for these variables, we adopt fuzzy logic as the evaluator, named the fuzzy evaluator. A detailed description about the fuzzy evaluator is provided in Section 5.3.

The reasons for adopting multi-objective evaluator are listed below. Firstly, federated learning with heterogeneity needs to consider multiple factors, including local computational capability, network bandwidth, training data, and the non-independent and identically distributedness of training data. These factors need simultaneous consideration to enhance performance. Secondly, in practical scenarios, these factors fluctuate with the location of vehicles, time, and environments. Therefore, assessing the vehicles locally is better than using a central server to do so. Thirdly, the relationship among these factors lacks explicit expressions, and they exhibit fuzzy relationships with each other. A multi-objective evaluator with fuzzy logic does not require explicit expressions and is easy to configure for any new scenarios. Based on the above, we adopt a multi-factor evaluator with fuzzy logic to assess the vehicles.

### 5.1. Prediction to the Network Throughput

The network environment can directly affect exchanging the model between the FL server and the clients. In general, the network throughput at some time can be predicted according to the historical transmitting state in the past.

Communication between the FL server and the client mainly consists of two parts in each round, specifically, broadcasting the global model and uploading the local model to the FL server. The time to broadcast the global model does not affect the performance of the FL since the time can be considered a constant in each round [35], and the constant does not change anytime or anywhere. The time to upload the local model is the main component in FL communication.

We consider reliable transmitting protocols, such as the transmission control protocol (TCP), used as the exchanging model protocol to upload the local model to the FL server. Therefore, TCP (Reno), a widely used protocol, is adopted to transmit the local model to ensure the trust and reliability of the model with the best effort.

The available throughput of the participating vehicles varies with the mobility of the vehicles. In practice, precisely predicting throughput is necessary for each participant when the fuzzy evaluator assesses the participating vehicle. To predict the throughput available, the sender’s congestion control (CWND_SND) window size in the TCP (RENO) is used to approximate the throughput of the participants. The assumptions are that every participating vehicle plays the sender’s role in sending the data to the FL server, and the history record of CWND_SND is stored in the sender when the data are transmitted. The available throughput of the participating vehicles achieves by averaging the CWND_SND values within a certain period.

The clarification is that the value of available throughput need not be exact, and the obtained value meets some criteria that keep the order, relatively. In other words, the order of the predicted throughput of the participating vehicles also keeps the order in terms of the real throughput in the real world since the evaluator only requires sorting the participating vehicles by the available throughput. In the real world, because of user privacy, predicting avaible throughput accurately has the difficulties.The congestion window size can reflect the variety of available throughput, while the network environment changes with mobility.

### 5.2. Training Time

Because of the characteristic of heterogeneity, participating vehicles owning the computational capability differ from each other. Meanwhile, the training dataset hardly distributes uniformly over participating vehicles in terms of the sample quantity and classification. The time spent in the training is not identical because every participant has a different computational capability and non-independent and identically distributed dataset (here, non-independent and identically distributed refers to the feature of independent and identically distributed). The drawback of the simulator is the time taken in training, which the client needs to learn previously. Moreover, the simulation process must show the heterogeneity of the FL system mentioned above. Hence, the time taken in the training is calculated by the equation below:

(6) $$T_{i}^{\mathrm{com}}=\frac{EC_{i}\left|D_{i}^{k}\right|}{B_{size}B_{exe}},$$

where $B_{size}$ refers to the batch size, and *E* refers to the number of epochs in the learning. $B_{size}$ and *E* are described as a constant and are the same for all participants in the FL learning process. $B_{exe}$ denotes the time to train the model on the client for $B_{size}$ samples. The value of $B_{exe}$ averages a real value, which is obtained from a huge amount of the experiments conducted on PyTorch [36]. Conducting experiments on the environments is described as follows. The hardware and software configurations are the Intel@Core™ i5 multi-core processor, CPU@2.50 GHz × 8 core, RAM@16 GiB, and PyTorch@1.8 version without GPU.

### 5.3. Fuzzy Evaluator with Multi-Objective

Fuzzy logic is an approach that does not need a close-form solution over considering the variables and can obtain the list of the output values, having the characteristic of the lightweight, fuzzy logic run on the participating vehicles. We consider four input variables, specifically, the sample quantity, throughput available, computational capability, and loss function of the dataset, which are related to the uploading model success rate as well as the contribution of the global model. These input variables are essential to evaluate whether a participating vehicle is “good” or “bad” for the FL. The reasons are listed as follows. On the one hand, the distance between the vehicle and BSes/RSU varies with the time domain, and the throughput also fluctuates. Similarly, the computational capability is also frequently changed over time. The two input variables above are the main factors affecting the uploading model’s success rate. On the other hand, the dataset with more samples contributes more to the convergence. Meanwhile, in the FL with the non-independent and identically distributed feature, the diversity of the dataset across the participating vehicles can accelerate the convergence and be measured by the loss function. The greater the loss function, the greater the diversity of the dataset [14]. Therefore, the sample quantity and the loss function are introduced to measure the quality of the dataset. Next, we present the input variables and their description.

**Figure 5:**
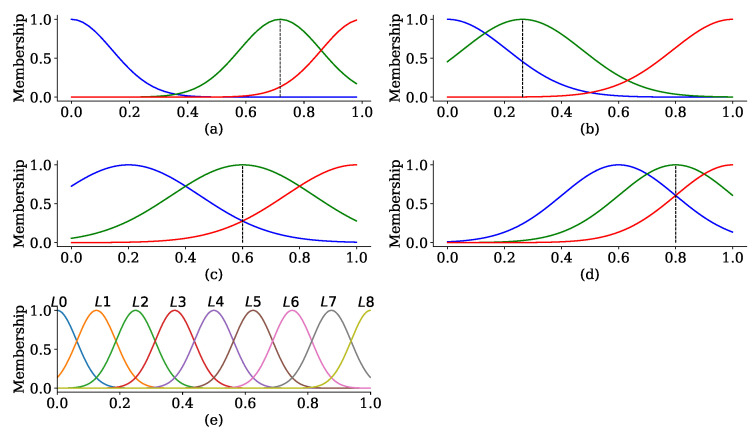
Membership functions used in the fuzzy evaluator. (**a**) Normalized sample quantity. (**b**) Normalized available network throughput. (**c**) Normalized computational capability on the local. (**d**) Normalized loss function training on the local dataset. (**e**) Evaluation mapped into different levels. In subfigure (**a**–**d**), the red line represents that the participant has better performance (or owns more resources) on a specific factor. The green/blue lines mean that the participant has average/poor performance (or owns average/less resource) on the specific factor. The dashed line in subfigure (**a**–**d**) represents the mean value of each variable.

Sample quantity (SQ):The convergence can be accelerated when more samples are trained in machine learning. Similarly to FL, the client with more samples participates in the FL, speeding up the convergence. Hence, the participant with more samples should be selected as the client to join the FL. Considering the number of clients, selecting as many clients as possible is equivalent to training more samples in the round. However, the number of clients must be restricted because of the bandwidth limitation. Selecting a client with more samples is more efficient than the method that selects many clients. Therefore, the fuzzy evaluator uses the sample quantity as an input variable. The value of the sample quantity is normalized into [0, 1]. The normalization is mapped into three levels—sufficient, average, and shortage—as shown in Figure 5a.

Throughput available (TA): The throughput determines whether the model is uploaded successfully. This variable represents the network environment of the vehicle and is affected by the number of nodes around, allocated RBs, and the distance to the BSes/RSU. Obtaining the throughput availability is described in Section 5.1. Figure 5b shows the membership function of the throughput availability. Similarly to the sample quantity, the throughput available is normalized and mapped into three levels of good, middle, and poor as shown in Figure 5b.

Computational capability (CC): The computational capability determines how fast the learning is. This variable denotes the available computing power of the participant, which is one of the factors affecting the training time. Similarly to the sample quantity, the computational capability is also normalized and mapped into three levels of strong, middle, and weak as shown in Figure 5c.

Loss function (LF):Training various samples can improve the generalization ability of the model. Selecting a client with diversity has more contribution to the convergence. In the paper, we adopt the loss function to measure the diversity of the dataset, calculated by (7):

(7) $$l_{i}=\frac{\sum_{i=1}^{|D_{i}^{k}|}L_{i}\left(w_{i}^{k},x_{i}^{k},y_{i}^{k}\right)}{|D_{i}^{k}|}$$

The loss function calculation is the same as the loss function in the training but without updating the gradient. In addition, all samples need to calculate the loss function once to average the error. The shuffling sample does not affect the result without updating the model. The greater loss function represents that the dataset owns higher diversity and some new features. The loss function is also normalized and mapped into three levels of greater, middle, and lower as shown in Figure 5d.

All variables adopt the Gaussian function as the membership function to ensure that a different input value results in different output for the evaluation. The dashed line represents the mean of the input variable calculated from the historical records.

For the unity of the expression, all input variables are normalized into [0, 1] by (8). Two variables named “Value” and “Maximumofinputvariable” need to be replaced by the actual value of a specific input variable in (8):

(8) $$Normalized=\frac{Value}{Maximum\;of\;input\;variable}\times 100\%.$$

where “*Value*” denotes the result of a specific input variable gathered locally in real time, as variables with different measure need to be mapped to the same scale. “*Maximum of input variable*” denotes the maximum value of an input variable in the historical record.

The fuzzy evaluator comprises four components: fuzzification, fuzzy rules, defuzzification, and client selection. The Mamdani method is used as the fuzzy inference technique. Regarding the fuzzification of the output value, the output of four variables is mapped into three levels as shown in Figure 5a–d. The normalization of the input variable is associated with the output variable through fuzzy rules and output to nine levels from $L_{0}$ to $L_{8}$. Detailed fuzzy rules are listed in Table 2.

Fuzzification is such that the crisp input value needs to be transformed into three different linguistics. These linguistics are described in Section 5.3. Moreover, the bound of each linguistic is defined through historical records.

The fuzzy rule contains 81 items since there are four input variables, and each input variable is mapped into three linguistics as shown in Table 2. Each item in Table 2 is implemented by a simple if–then logic with single or multiple antecedents. Finally, all antecedents are outputted to one consequent. Those rules are essential to the evaluation of participating vehicles. Many experiments are conducted to decide the mapping relationship between the input and output, and the experiment with the best performance is selected as the item of the fuzzy rule.

**Figure 6:**
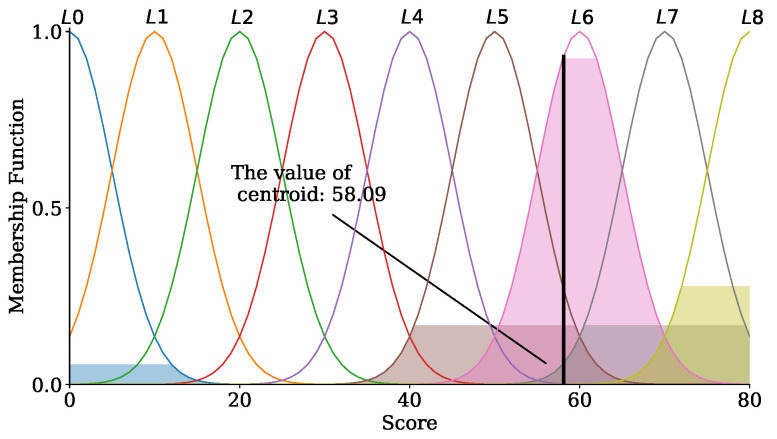
The center of gravity (COG).

Defuzzification is that the output needs to be transformed to a scalar through the center of gravity (COG), one of the most commonly used methods. COG is defined as shown in (9):

(9) $$y^{*}=\frac{\sum_{i=1}^{n}a_{i}\mu \left(a_{i}\right)}{\sum_{i=1}^{n}\mu \left(a_{i}\right)}$$

where $a_{i}$, $\mu (a_{i})$, and *n* denote the sample element, the membership function, and the number of the element in the group, respectively. Figure 6 illustrates the COG. The value of 58.09 in Figure 6 represents an output calculated by (9), and the value belongs to the L6 level.

The final evaluation is broadcast over DSRC communication to all neighbors. Each participating vehicle maintains a table to store and update the evaluation received from the neighbors and itself. Ultimately, the table’s top *m* contains its id. The participating vehicle becomes a client, while the table contains its id and vice versa. Following this, the selected client trains the dataset and uploads their local model.

## 6. Simulation and Evaluation

### 6.1. Set Up

A simulator is constructed to simulate realistic wireless vehicular networks, integrating with OMNeT for communication [37], simuLTE for the cellular network [38], SUMO for vehicular mobility [39], and Pytorch for federated learning [36]. The number of vehicles is set to 30. All vehicles are running on a straight road with 1000 m in length and follow the free-way model. Each vehicle has two communication interfaces: cellular and DSRC (like the IEEE 802.11p interface). In the network, multiple BSes are located uniformly on the map, allocating wireless resources to the vehicle. Wireless resources are allocated to up/downlink streams identically. The available throughput of the participating vehicle reaches 10.4 Mbps when enjoying the highest modulation coding scheme (MCS) and the whole wireless resources. On the contrary, the available throughput only reaches 0.24 Mbps under the lowest MCS and the whole wireless resources. The evaluation of the participating vehicle is broadcast to the neighboring vehicles over the IEEE 802.11p interface in a fixed interval. Each participating vehicle needs to maintain a table which stores the evaluation of the neighbor in the specific range. Furthermore, the content of the table is also updated in fixed intervals. To avoid the occurrence of stragglers, the deadline of the communication round is introduced in the simulator and set to 20 s. The local model received after the deadline is discarded.

A dataset regarding image recognition, MNIST [40], is adopted as the training dataset. To meet the characteristic of non-independent and identically distributed, we synthesize the dataset with the non-independent and identically distributed feature and the whole sample in MNIST is re-distributed over the participating vehicles according to the following rules. The local dataset of each vehicle can come from multiple classes which own identical quantity samples. These vehicles have an unbalanced dataset. For example, the vehicle’s id numbers from 0 to 11 have about 4500 samples, while the other vehicle’s id numbers from 12 to 29 only own about 45 samples. All samples in the participating vehicles are not duplicates of each other. Considering the vehicle density running on the road, the number of selected clients in each range of 200 m is up to 2. In the centralized client selection, the FL server selects five clients in each round. The parameters used in the simulator are listed in Table 3.

The learning model used in the FL has seven layers, including two convolution layers, one flattened layer, two max pooling layers, and two fully connected layers, to train the MNIST dataset. Each sample has a size of 28 × 28 and single channels. The total number of the trainable variables in the learning model is about 1.66 million and takes the disk space to 5.2 Mbytes. All models are not compressed in the broadcasting and uploading stage.

### 6.2. Evaluation

In this subsection, we evaluate the performance compared to the baselines and the proposal in terms of the model accuracy, the influence on the distribution of the vehicle, the convergence over the non-independent and identically distributed dataset and the accumulated consumed time on the communication overhead.

To evaluate the performance of the proposal, we consider multiple benchmarks, specifically, centralized client selection (CCS) and centralized client selection with fuzzy logic (CCS-fuzzy) [8]. CCS means that all information involving the active states of the client needs to be transmitted to the FL server, and the FL server is in charge of the client selection. Random client selection is a typical CCS scheme. CCS-fuzzy refers to the fuzzy evaluation to assess the client being moved from the FL server to the participating vehicles and uploaded to the FL server after evaluation. DCS refers to selecting a client which does not rely on the FL server. The evaluation of the participant is exchanged among the neighbors over DSRC communication. These nodes elect some nodes with the highest evaluation to be acted as the clients for uploading the model.

In the CCS-fuzzy and random scheme, the FL server selects five clients randomly from all vehicles. Some clients may become the stragglers in the selected clients. In general, the straggler cannot upload their local model before the deadline expires because their computational capability and network throughput are too low.

**Figure 7:**
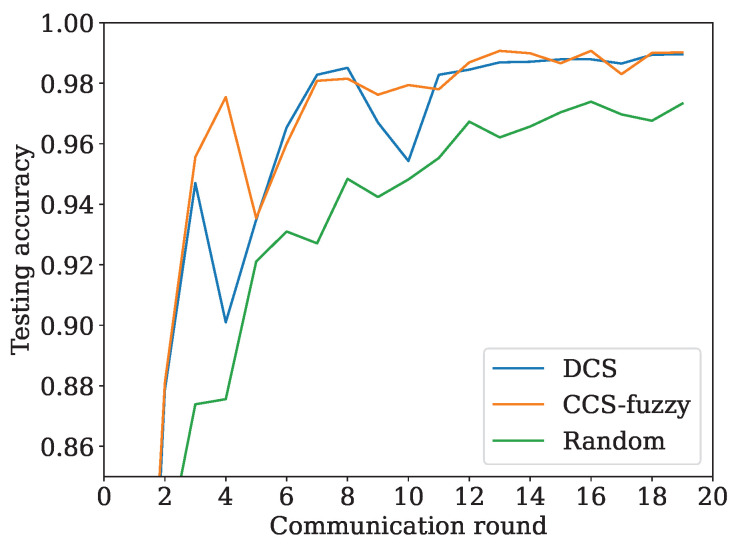
Accuracy of DCS, CCS-fuzzy, and random scheme.

Figure 7 compares the accuracy of DCS, random scheme, and CCS-fuzzy. The sample quantity of the vehicle is the same as in Table 3. Every vehicle owns nine classes, each containing an identical sample quantity. The number of selected clients in the DCS is averaged at 5.15. The number of selected clients in the random scheme and CCS-fuzzy is set to five as a constant. The results can be observed from Figure 7 that the CCS-fuzzy outperforms DCS and the random scheme. The reason is that CCS-fuzzy can select the participating vehicle with the highest evaluation as the client and can accelerate the convergence. In other words, CCS-fuzzy can choose clients with better resources in terms of computational, communication and local datasets. However, it is notable that the proposal also performs well. The two curves of CCS-fuzzy and the proposed scheme are overlapped in the final stage, while DCS largely jitters in the initial step. In conclusion, DCS can achieve the same level as CCS-fuzzy. Furthermore, DCS can outperform the random scheme after a certain communication round.

**Figure 8:**
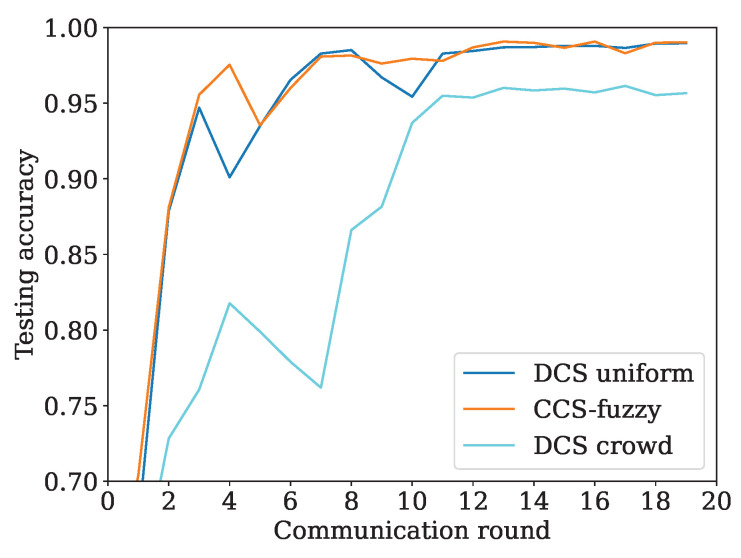
Different vehicle distributions influence the accuracy.

The distribution of participating vehicles can impact the performance of DCS because DCS can select the optimal client in the neighboring small area. To illustrate the case, we design two distributions for participating vehicles, including uniform distribution and extreme distribution, respectively. In uniform distribution, all vehicles are distributed randomly. In the extreme distribution, the vehicles with better evaluation are crowded in one small area, while the remaining vehicles with poor evaluation are crowded in another small area. In Figure 8, the results illustrate the performance involving CCS-fuzzy, DCS with uniform distribution, and DCS with extreme distribution. Other parameters are the same as in Table 3. The number of selected clients in the uniform distribution is averaged at 5.05, while the number of selected clients in the extreme distribution is 6 as a constant. The observation from Figure 8 is that the accuracy of the uniform distribution is approaching the CCS-fuzzy scheme and is better than the performance of the extreme distribution. The reason is described as follows. The vehicles with better evaluation are more likely to be selected as the client in the uniform distribution. On the contract, the vehicles with better evaluation are crowded, leading to “cut-throat competition” in the extreme distribution. Hence, a small number of vehicles with better evaluation are selected as the client and pull down the convergence speed.

**Figure 9:**
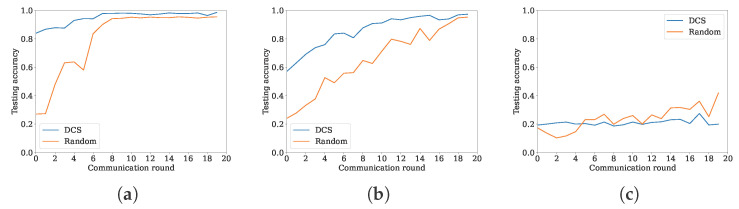
Non-independent and identically distributed characteristic impacts on the accuracy. (**a**) Client owns 9 of 10 classes. (**b**) Each client owns 6 of 10 classes. (**c**) Each client owns 2 of 10 classes.

To show the performance regarding the non-independent and identically distributed dataset, we run three experiments with an unbalanced quantity dataset, in which each vehicle contains 9 classes, 6 classes, and 2 classes of 10 classes, respectively. Figure 9 compares DCS and random scheme over the non-independent and identically distributed dataset. In Figure 9, the DCS scheme refers to DCS-uniform, specifically the vehicles distributed uniformly. Under this scenario, the curves of the DCS and CCS-fuzzy schemes overlap each other, as the two schemes select the same nodes as shown in Figure 8. In Figure 9a–c, the number of selected clients in DCS is averaged at 5.15, 5.2 and 4.95, respectively. The number of selected clients for the random scheme is set to 5 as a constant. The conclusion from Figure 9a–c observed is that the non-independent and identically distributed characteristic of the dataset has a great impact on the model accuracy and the convergence speed. The convergence speed accelerates when the characteristics of the dataset are approaching from non-independent and identically distributed to independent and identically distributed because the non-independent and identically distributed dataset increases the weight shifting in training. In the special case, such as without the intersection between the datasets, the accuracy cannot meet the requirements and even cannot converge as shown in Figure 9c. On the other hand, the proposal performs well compared to the random scheme but is extremely non-independent and identically distributed. The reason is that the loss function of local data is considered in the client selection stage. It makes to enhance the diversity of the dataset and decreases the non-independent and identically distributed characteristics.

**Figure 10:**
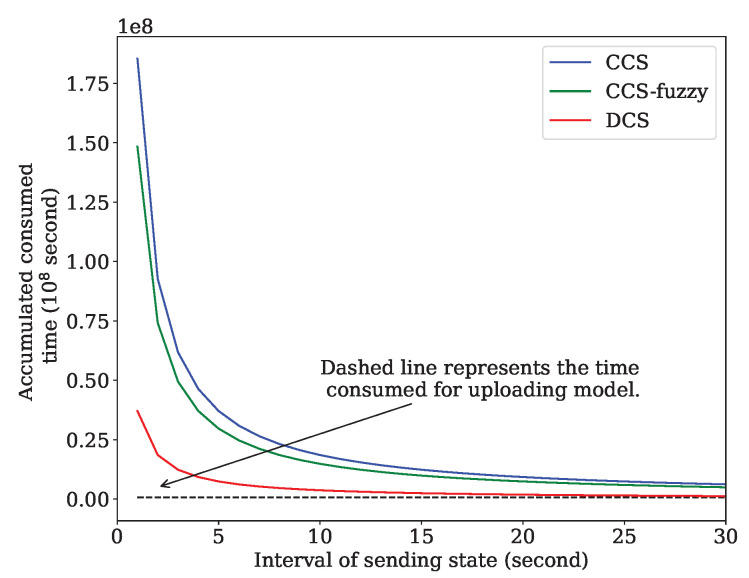
Accumulated consumed time vs. sending interval.

Finally, we analyze the time consumed on the communication overhead. We adopt the accumulated consumed time as a metric, the sum of time each participant consumed on the communication. We adopt Tokyo region as an example to analyze the communication overhead. According to the statistics [41], by 2021, the number of registered motor vehicles reached 3.09 million in Tokyo region, Japan. In each communication round, 1000 vehicles are selected as the client. We consider the sum of the total time consumed in communication, including exchanging the model and maintaining the active state of the vehicles. Sending an active state also spends a full latency since it is considered a small packet. Other parameters are listed in Table 3. Figure 10 compares the accumulated consumed time over DCS, CCS-fuzzy, CCS and exchanging the model, except for the random scheme, as it does not need any state information of the vehicle in the selection process. So, in the random scheme, the overhead of the exchange information is zero. The dashed line represents the consumed time for the exchanging of the model. The following results can be seen in Figure 10. First, compared to exchanging the model, the cost caused by maintaining an active state cannot be neglected when the number of participants increases drastically. This conclusion is ignored by most of the prior researchers. Second, accumulated consumed time decreases with the increase in the sending interval, but maintaining an active state also consumes enormous time and energy in CCS and CCS-fuzzy. In addition, the time consumed by DCS is less than that of CCS and CCS-fuzzy. The reasons are as follows. The vehicle-to-vehicle latency using DSRC communication is smaller than the latency from the vehicle to the cloud. Additionally, the multi-objective evaluator running locally not only compresses all the information but protects privacy by avoiding sending all the information involving the vehicle to the neighbors. Finally, broadcasting the evaluation is restricted in each small area, such as a range of 200 m, and reduces the communication overhead.

## 7. Conclusions

In this paper, we propose a novel client selection scheme, namely, distributed client selection with a multi-objective evaluator, in which the FL server is not in charge of client selection and does not gather information from the participating vehicles. Extensive simulations conducted show that the overhead of exchanging state information between the vehicle and the central server is not negligible, especially when the number of nodes reaches hundreds of millions. Secondly, the optimization of selecting nodes still faces challenges, as there is no closed-form solution for multiple factors. The heterogeneity of samples across vehicles further increases the difficulties of federated learning.

## Figures and Tables

**Table 1:** The parameters referred from Gboard [5].

Parameters	Value
The number of whole device	1.5 million
The period of a communication round	72 s
Model size	1.4 Million byte
The number of selected client in each round (average)	300
The size of active state (CFL)	100 byte
The size of active state (CFL-fuzzy)	30 byte

**Table 2:** Parts of fuzzy rule.

	SQ	TA	CC	LF	Evaluation
1	sufficient	high	strong	greater	L8
2	average	high	strong	greater	L7
3	shortage	high	strong	greater	L6
…	…	…	…	…	…
52	sufficient	poor	weak	middle	L2
53	average	poor	weak	middle	L1
54	shortage	poor	weak	middle	L0
…	…	…	…	…	…
79	sufficient	poor	weak	smaller	L0
80	average	poor	weak	smaller	L0
81	shortage	poor	weak	smaller	L0

SQ: sample quantity, TA: throughput available, CC: computational capability, LF: loss function.

**Table 3:** Configuration of the simulator for distributed client selection.

Parameters	Value
RBs of upstream/downstream	1:1
Batch size	20 sample
Epochs	30
Execution time of one batch $B_{exe}$	0.06 s
Highest throughput (cellular network)	10.4 Mbps
Worst throughput (cellular network)	0.24 Mbps
Range of exchanging evaluation over DSRC	200 m
The number of vehicles	30
Deadline of communication round	20 s
The number of selected clients in each area (distributed)	2
The length of road	1000 m; straight road
The location of the FL server	At 520 m of the road (very close to the BS)
The sample quantity of the vehicle	VehID 0–11: about 4500 images, VehID 12–29: about 45 images
The vehicle distribution	uniform
The packet size	1500 byte
The latency from vehicle to cloud	200 ms
The latency from vehicle to vehicle (DSRC)	40 ms

## Data Availability

There is no data availability for the paper.

## References

[1] McMahan B., Moore E., Ramage D., Hampson S., y Arcas B.A. (2017). Communication-efficient learning of deep networks from decentralized data. Artificial Intelligence and Statistics.

[2] Kairouz P., McMahan H.B., Avent B., Bellet A., Bennis M., Bhagoji A.N., Bonawitz K., Charles Z., Cormode G., Cummings R. (2021). Advances and open problems in federated learning. Found. Trends^®^ Mach. Learn..

[3] Posner J., Tseng L., Aloqaily M., Jararweh Y. (2021). Federated learning in vehicular networks: Opportunities and solutions. IEEE Netw..

[4] Hammoud A., Otrok H., Mourad A., Dziong Z. (2022). On Demand Fog Federations for Horizontal Federated Learning in IoV. IEEE Trans. Netw. Serv. Manag..

[5] Hard A., Rao K., Mathews R., Beaufays F., Augenstein S., Eichner H., Kiddon C., Ramage D. (2018). Federated Learning for Mobile Keyboard Prediction. arXiv.

[6] Nishio T., Yonetani R. Client Selection for Federated Learning with Heterogeneous Resources in Mobile Edge. Proceedings of the IEEE International Conference on Communications (ICC).

[7] Abdulrahman S., Tout H., Mourad A., Talhi C. (2021). FedMCCS: Multicriteria Client Selection Model for Optimal IoT Federated Learning. IEEE Internet Things J..

[8] Cha N., Du Z., Wu C., Yoshinaga T., Zhong L., Ma J., Liu F., Ji Y. (2022). Fuzzy Logic Based Client Selection for Federated Learning in Vehicular Networks. IEEE Open J. Comput. Soc..

[9] Hamer J., Mohri M., Suresh A.T. Fedboost: A communication-efficient algorithm for federated learning. Proceedings of the International Conference on Machine Learning.

[10] Konečnỳ J., McMahan H.B., Yu F.X., Richtárik P., Suresh A.T., Bacon D. (2016). Federated learning: Strategies for improving communication efficiency. arXiv.

[11] WANG L., WANG W., LI B. CMFL: Mitigating Communication Overhead for Federated Learning. Proceedings of the 2019 IEEE 39th International Conference on Distributed Computing Systems (ICDCS).

[12] Niknam S., Dhillon H.S., Reed J.H. (2020). Federated Learning for Wireless Communications: Motivation, Opportunities and Challenges. arXiv.

[13] Almanifi O.R.A., Chow C.O., Tham M.L., Chuah J.H., Kanesan J. (2023). Communication and computation efficiency in Federated Learning: A survey. Internet Things.

[14] Cho Y.J., Wang J., Joshi G. (2022). Towards understanding biased client selection in federated learning. Proceedings of the International Conference on Artificial Intelligence and Statistics.

[15] Wang H., Kaplan Z., Niu D., Li B. Optimizing Federated Learning on Non-IID Data with Reinforcement Learning. Proceedings of the IEEE INFOCOM 2020—IEEE Conference on Computer Communications.

[16] Zhang P., Wang C., Jiang C., Han Z. (2021). Deep Reinforcement Learning Assisted Federated Learning Algorithm for Data Management of IIoT. IEEE Trans. Ind. Inform..

[17] Zhou X., Ye X., Wang K.I.K., Liang W., Nair N.K.C., Shimizu S., Yan Z., Jin Q. (2023). Hierarchical Federated Learning With Social Context Clustering-Based Participant Selection for Internet of Medical Things Applications. IEEE Trans. Comput. Soc. Syst..

[18] Roy A.G., Siddiqui S., Pölsterl S., Navab N., Wachinger C. (2019). Braintorrent: A peer-to-peer environment for decentralized federated learning. arXiv.

[19] Li Y., Chen C., Liu N., Huang H., Zheng Z., Yan Q. (2020). A blockchain-based decentralized federated learning framework with committee consensus. IEEE Netw..

[20] Lu Y., Huang X., Zhang K., Maharjan S., Zhang Y. (2020). Blockchain Empowered Asynchronous Federated Learning for Secure Data Sharing in Internet of Vehicles. IEEE Trans. Veh. Technol..

[21] Pokhrel S.R., Choi J. A decentralized federated learning approach for connected autonomous vehicles. Proceedings of the 2020 IEEE Wireless Communications and Networking Conference Workshops (WCNCW).

[22] Ye H., Liang L., Li G.Y. (2022). Decentralized federated learning with unreliable communications. IEEE J. Sel. Top. Signal Process..

[23] Fu L., Zhang H., Gao G., Zhang M., Liu X. (2023). Client Selection in Federated Learning: Principles, Challenges, and Opportunities. IEEE Internet Things J..

[24] AbdulRahman S., Tout H., Ould-Slimane H., Mourad A., Talhi C., Guizani M. (2020). A survey on federated learning: The journey from centralized to distributed on-site learning and beyond. IEEE Internet Things J..

[25] Shi Y., Yu H., Leung C. (2023). Towards fairness-aware federated learning. IEEE Trans. Neural Netw. Learn. Syst..

[26] Ezzeldin Y.H., Yan S., He C., Ferrara E., Avestimehr A.S. Fairfed: Enabling group fairness in federated learning. Proceedings of the AAAI Conference on Artificial Intelligence.

[27] Xu J., Wang H. (2021). Client Selection and Bandwidth Allocation in Wireless Federated Learning Networks: A Long-Term Perspective. IEEE Trans. Wirel. Commun..

[28] Huang T., Lin W., Wu W., He L., Li K., Zomaya A.Y. (2021). An Efficiency-Boosting Client Selection Scheme for Federated Learning With Fairness Guarantee. IEEE Trans. Parallel Distrib. Syst..

[29] Li T., Sahu A.K., Zaheer M., Sanjabi M., Talwalkar A., Smith V. (2020). Federated optimization in heterogeneous networks. Proc. Mach. Learn. Syst..

[30] Wang S., Liu F., Xia H. Content-based vehicle selection and resource allocation for federated learning in IoV. Proceedings of the 2021 IEEE Wireless Communications and Networking Conference Workshops (WCNCW).

[31] Zhang X., Mavromatics A., Vafeas A., Nejabati R., Simeonidou D. (2023). Federated Feature Selection for Horizontal Federated Learning in IoT Networks. IEEE Internet Things J..

[32] Karimireddy S.P., Kale S., Mohri M., Reddi S., Stich S., Suresh A.T. Scaffold: Stochastic controlled averaging for federated learning. Proceedings of the International Conference on Machine Learning.

[33] Manias D.M., Shami A. (2021). Making a Case for Federated Learning in the Internet of Vehicles and Intelligent Transportation Systems. IEEE Netw..

[34] Hu X., Li R., Ning Y., Ota K., Wang L. (2023). A Data Sharing Scheme Based on Federated Learning in IoV. IEEE Trans. Veh. Technol..

[35] Xu H., Li J., Xiong H., Lu H. FedMax: Enabling a Highly-Efficient Federated Learning Framework. Proceedings of the 2020 IEEE 13th International Conference on Cloud Computing (CLOUD).

[36] Paszke A., Gross S., Massa F., Lerer A., Bradbury J., Chanan G., Killeen T., Lin Z., Gimelshein N., Antiga L. Pytorch: An imperative style, high-performance deep learning library. Proceedings of the Advances in Neural Information Processing Systems.

[37] Varga A., Hornig R. An overview of the OMNeT++ simulation environment. Proceedings of the 1st International ICST Conference on Simulation Tools and Techniques for Communications, Networks and Systems.

[38] Virdis A., Stea G., Nardini G. SimuLTE-A modular system-level simulator for LTE/LTE-A networks based on OMNeT++. Proceedings of the 2014 4th International Conference On Simulation And Modeling Methodologies, Technologies and Applications (SIMULTECH).

[39] Lopez P.A., Behrisch M., Bieker-Walz L., Erdmann J., Flötteröd Y.P., Hilbrich R., Lücken L., Rummel J., Wagner P., Wießner E. Microscopic traffic simulation using sumo. Proceedings of the International Conference on Intelligent Transportation Systems (ITSC).

[40] Deng L. (2012). The mnist database of handwritten digit images for machine learning research. IEEE Signal Process. Mag..

[41] Number of Registered Motor Vehicles in Tokyo, Japan from 2012 to 2021. https://www.statista.com/statistics/1191244/japan-number-motor-vehicles-in-use-tokyo/.

